# MicroRNA-155 targets SOCS1 to inhibit osteoclast differentiation during orthodontic tooth movement

**DOI:** 10.1186/s12903-023-03443-8

**Published:** 2023-12-01

**Authors:** Yao Jiao, Sicong Mi, Xiaoyan Li, Yitong Liu, Nannan Han, Junji Xu, Yi Liu, Song Li, Lijia Guo

**Affiliations:** 1 Department of Periodontics, School of Stomatology, Capital Medical University, Tian Tan Xi Li No.4, Beijing, 100050 P. R. China; 2 Department of Stomatology, Air Force Medical Center, PLA, The Fourth Military Medical University, Beijing, 100142 P. R. China; 3 Laboratory of Tissue Regeneration and Immunology, Department of Periodontics, Beijing Key Laboratory of Tooth Regeneration and Function Reconstruction, School of Stomatology, Capital Medical University, Beijing, 100050 P. R. China; 4 Department of Orthodontics, School of Stomatology, Capital Medical University, Tian Tan Xi Li No.4, Beijing, 100050 P. R. China; 5 Department of Orthodontics (WangFuJing Campus), School of Stomatology, Capital Medical University, Scylla alley No.11, Beijing, 100006 P. R. China

**Keywords:** MicroRNA-155, Osteoclast, Orthodontics, Monocyte

## Abstract

**Background:**

MicroRNA-155 (miR-155) is a multifunctional miRNA whose expression is known to be involved in a range of physiological and pathological processes. Its association with several oral diseases has been established. However, the specific role of miR-155 in orthodontic tooth movement remains unclear. In this study, we investigated the impact of miR-155 on osteoclast differentiation and orthodontic tooth movement models, aiming to explore the underlying mechanisms.

**Methods:**

In this experiment, we utilized various agents including miR-155 mimic, miR-155 inhibitor, as well as non-specific sequences (NC mimic & NC inhibitor) to treat murine BMMNCs. Subsequently, osteoclast induction (OC) was carried out to examine the changes in the differentiation ability of monocytes under different conditions. To assess these changes, we employed RT-PCR, Western blotting, and TRAP staining techniques. For the orthodontic tooth movement model in mice, the subjects were divided into two groups: the NaCl group (injected with saline solution) and the miR-155 inhibitor group (injected with AntagomiR-155). We observed the impact of orthodontic tooth movement using stereoscopic microscopy, micro-CT, and HE staining. Furthermore, we performed RT-PCR and Western blotting analyses on the tissues surrounding the moving teeth. Additionally, we employed TargetScan to predict potential target genes of miR-155.

**Results:**

During osteoclast induction of BMMNCs, the expression of miR-155 exhibited an inverse correlation with osteoclast-related markers. Overexpression of miR-155 led to a decrease in osteoclast-related indexes, whereas underexpression of miR-155 increased those indexes. In the mouse orthodontic tooth movement model, the rate of tooth movement was enhanced following injection of the miR-155 inhibitor, leading to heightened osteoclast activity. TargetScan analysis identified SOCS1 as a target gene of miR-155.

**Conclusions:**

Our results suggest that miR-155 functions as an inhibitor of osteoclast differentiation, and it appears to regulate osteoclasts during orthodontic tooth movement. The regulatory mechanism of miR-155 in this process involves the targeting of SOCS1.

**Supplementary Information:**

The online version contains supplementary material available at 10.1186/s12903-023-03443-8.

## Background

Orthodontic tooth movement occurs as a result of mechanical force exerted on the teeth, leading to remodeling of the periodontal membrane and alveolar bone. When the appropriate orthodontic force is applied, the periodontal membrane transmits this force to the alveolar bone. This transmission of force causes bone resorption on the side experiencing pressure. It is widely recognized that this step is considered the rate-limiting step in orthodontic tooth movement [1]. Enhanced alveolar bone resorption, facilitated by osteoclasts, can expedite the process of orthodontic tooth movement and reduce the duration of orthodontic treatment [2]. Currently, the mechanism underlying orthodontic tooth movement remains incompletely understood. Researchers have proposed a division of the roles played by periodontal ligament cells and bone cells into four distinct stages throughout the process of orthodontic tooth movement. These stages include cell external biology, cell strain, cell activation and differentiation, and tissue reconstruction of the periodontal tissue [3]. Apart from intercellular signaling, certain non-coding RNAs also contribute significantly to gene expression and translation during the cell strain phase [4].

MicroRNAs (miRNAs) are a group of non-coding small RNA molecules, typically composed of 20–22 nucleotides. They play a crucial role in post-transcriptional gene regulation by targeting specific genes. MiRNAs bind to the 3’-untranslated region (3’-UTR) of target genes, resulting in reduced gene expression at the post-transcriptional level. This binding can lead to mRNA degradation or transcriptional inhibition, thereby regulating post-transcriptional gene expression [5]. MicroRNAs have emerged as crucial regulators in numerous biological processes, encompassing cell development, cell differentiation, cell proliferation, cell cycle regulation, and metabolism [6–8]. To date, the discovery of human miRNAs has exceeded 2000 [9], with more than 3% of genes identified as coding for miRNAs. It has been estimated that miRNA-mediated gene regulation affects approximately 40–90% of human protein-coding genes [10]. Furthermore, miRNAs, as mechanosensitive non-coding RNAs, fulfill significant functions in the regulation of bone remodeling and the facilitation of osteoblast and osteoclast differentiation [11]. Studies have indicated that alterations in the expression of multiple miRNAs in saliva and gingival crevicular fluid of orthodontic patients can serve as biomarkers for tooth movement. In the future, it is suggested that targeted modulation of miRNA expression locally could potentially lead to a reduction in orthodontic treatment duration and mitigate side effects such as root resorption, decreased risk of caries, and gingival inflammation [12].

MicroRNA-155 (miR-155) is a multifunctional miRNA known to be involved in a wide range of physiological and pathological processes. Its expression changes have been associated with the regulation of hematopoietic lineage differentiation, immune response, inflammation, tumor development, viral infections, cardiovascular diseases, Down syndrome, and more [13, 14]. Currently, miR-155 has been established as being implicated in numerous oral diseases. The expression level of miR-155 in the peripheral blood of patients with oral squamous cell carcinoma (OSCC) is higher compared to that of healthy individuals. Consequently, miR-155 can serve as a diagnostic biomarker for oral squamous cell carcinoma [15]. Furthermore, miR-155 exhibits high expression levels in the saliva of individuals with periodontitis. Its expression is positively associated with the severity of periodontitis and clinical indicators, indicating the potential involvement of miR-155 in the onset and progression of periodontitis [16]. In the gingival crevicular fluid of orthodontic patients experiencing root resorption, there is a decrease in the expression of miR-155 as the degree of orthodontic root resorption increases. In vitro experiments involving the induction of osteoclast differentiation in RAW264.7 cells demonstrated that the transfection of miR-155 mimics notably suppressed the formation of osteoclasts. Conversely, the transfection of miR-155 inhibitor significantly increased the formation of osteoclasts. These findings indicate the inhibitory role of miR-155 in osteoclast differentiation [17].

Currently, some researchers have demonstrated the inhibitory effect of miR-155 on the regulation of osteoclast differentiation [18]. However, the precise role of miR-155 in orthodontic tooth movement and its involvement in the tooth movement process remain unknown. As osteoclast differentiation is the rate-limiting step in orthodontic treatment, understanding the impact of miR-155 on osteoclast differentiation could potentially enhance the efficiency of orthodontic treatment. Therefore, this study aims to investigate the effect of miR-155 on osteoclast differentiation through in vitro experiments. Subsequently, a mouse orthodontic model has been established to explore the role and mechanism of miR-155 in orthodontic tooth movement and identify potential targets of miR-155. These findings may offer new prospects for shortening the duration of orthodontic treatment in the future.

## Methods

### Cell culture and osteoclast induction (OC)

Bone marrow-derived monocytes (BMMNCs) were obtained from 6-week-old male C57BL/6 mice and cultured. The experiments were conducted in accordance with the ARRIVE (Animal Research: Reporting of In Vivo Experiments) guidelines and the approved institutional guidelines for animal research set by the Animal Care and Use Committee of the Beijing Stomatological Hospital, Capital Medical University, Beijing, China (KQYY-202008-005). Mice were euthanized by inhalation of an overdose of isoflurane, and their bodies were immersed in 75% alcohol for 10 min. The femur and tibia from the lower limbs were isolated and washed with Phosphate Buffered Saline (PBS) containing Penicillin-Streptomycin solution (PS). The marrow was flushed out with medium until the marrow cavity turned white. The collected suspension was centrifuged at 1100 rpm for 6 min, and the supernatant was discarded. The cells were resuspended in cell suspension by adding 20% FBS α-MEM medium. The cell suspension was then incubated at 37 °C and 5% CO2 for 24 h. After incubation, the supernatant was aspirated from the culture dish. The cells were seeded in 6-well plates at a density of 1–2 × 10^6^ cells per well, using α-MEM medium supplemented with 10% FBS and 30ng/ml macrophage colony-stimulating factor (M-CSF) (PeproTech, USA). After 2 days of culture at 37 °C and 5% CO2, the BMMNCs appeared as small and round cells under the microscope. Osteoclastogenesis was initiated by adding receptor activator of nuclear factor-κB ligand (RANKL) at a concentration of 100ng/ml (PeproTech, USA) to the medium.

### Transfection of miR-155

All miR-155 transfection reagents used in this experiment were purchased from RiboBio (Guangzhou, China) Co., Ltd. A volume of 5 µl of 20µM miR-155 mimic and NC mimic was diluted in 120 µl of 1X riboFECT™ CP Reagent Buffer. For miR-155 inhibitor and NC inhibitor, a volume of 10 µl of 20µM stock solutions was gently mixed and diluted. Subsequently, a volume of 12 µl of riboFECT™ CP Reagent was added to the mixture, gently blown and mixed, and incubated at room temperature for 0–15 min. The resulting mixture was then added to 6-well plates containing PS-free medium to initiate cell transfection. The final concentration of the miRNA mimic was 50nM, and the final concentration of the miRNA inhibitor was 100nM. After 48 h, the total RNA of the cells was extracted. Some cells were switched back to 10% FBS α-MEM medium, and osteoclast-inducing factors (M-CSF 30ng/ml, RANKL 100ng/ml) were added for 3 or 5 days, during which the medium was changed once.

### Establishment of an animal model for orthodontic tooth movement

Twenty-four 6-week-old C57BL/6 male mice, weighing approximately 20 g, were obtained from SPF (Beijing) Biotechnology Co., Ltd. All experiments were conducted in accordance with animal ethics guidelines. The mice were randomly divided into four groups: TM 5d + NaCl (5 days of orthodontics tooth movement with NaCl injection), TM 12d + NaCl (12 days of orthodontics tooth movement with NaCl injection), TM 5d + AntagomiR-155 (5 days of orthodontics tooth movement with AntagomiR-155 injection), and TM 12d + AntagomiR-155 (12 days of orthodontics tooth movement with AntagomiR-155 injection) (n = 6/group). Three days before and on the day of orthodontic treatment, submucosal injections of 50 µl normal saline or 10nmol AntagomiR-155 dissolved in 50 µl saline were administered at four sites around the right maxillary first molar: buccal mesial-distal and palatal mesial-distal sites. During the second administration, the force was applied to the right maxillary first molar using a spring fixed to the upper incisor, which served as the anchorage teeth to mesially move the right maxillary first molar. At the end of the force application, the maxilla or the gingiva surrounding the mobile teeth was harvested from the animal model for subsequent analysis.

### Measurement of tooth movement distance

After the animals were euthanized, the maxillary specimens were collected and fixed in a 4% paraformaldehyde solution. The tooth movement distance of the first molar in each specimen was observed using stereoscopic microscopy. The Image J software was utilized to measure the shortest distance between the first molar and the second molar under the microscope. Three measurements of the same sample, performed by the same researcher, were averaged.

Micro-CT scanning was conducted on the tissue samples from various groups at the Medical Imaging Research and Testing Laboratory of Capital Medical University. Data Viewer, CT Vox, and CT An software were employed to measure the tooth movement distance. Specifically, the shortest distance between the crowns of the first and second molars in the sagittal direction was measured. Three measurements of the same sample, performed by the same researcher, were averaged.

### Haematoxylin and eosin (HE) staining

The animal tissues were decalcified using 10% EDTA in PBS (pH 8.0) at 4℃. After decalcification, the samples were dehydrated and embedded in paraffin. Sections of 5 μm thickness were obtained and stained with hematoxylin and eosin. The alveolar bone was examined using a light microscope (Media Cybernetics, USA).

### Tartrate-resistant acid phosphate (TRAP) staining

TRAP staining was performed on tissue sections from different groups in vivo and on osteoclasts in vitro after 5 days of induction. The staining procedure followed the standard protocol of the TRAP staining kit (Solarbio, China). Osteoclasts were identified as TRAP-positive cells with three or more nuclei, displaying a purplish-red color under the microscope. Following staining, five random fields were selected from each group for observation and counting using a light microscope (OLYMPUS, Japan). Statistical analysis was conducted to analyze the results.

### Real-time reverse transcriptasepolymerase chain reaction (real-time RTPCR)

Three days after osteoclast induction of BMMNCs, total RNA was extracted from the cells using Trizol reagent (Cwbio, China). The gingival tissue from the animal model was also isolated using Trizol reagent after sufficient grinding. Reverse transcription was carried out following the manufacturer’s protocol (Takara Biotechnology, Japan). RT-PCR reactions were performed using the SYBR Green PCR kit (Takara Biotechnology, Japan). The primer sequences for osteoclast-related markers are listed in Table 1. The primer sequences for mmu-miR-155-5p and U6 were provided by RiboBio (Guangzhou, China) Co., Ltd.

**Table 1:** Primer sequence

Target	Forward primer	Reverse primer
GAPDH	5’-ACCCAGAAGACTGTGGATGG-3’	5’-GGATGCAGGGATGATGTTCT-3’
NFATc1	5’-TGGCTACCGACATGTGTTGT-3’	5’-CTGGGGTTCTCCATCTGTGT-3’
TRAP	5’-GATGACTTTGCCAGTCAGCA-3’	5’-AACTGCTTTTTGAGCCAGGA-3’
CTSK	5’-TTCTCCTCTCGTTGGTGCTT-3’	5’-AAAAATGCCCTGTTGTGTCC-3’
SOCS1	5’-ACTTCTGGCTGGAGACCTCA-3’	5’-CCCAGACACAAGCTGCTACA-3’

### Western blotting analysis

Cells and gingival tissues were lysed using radioimmunoprecipitation (RIPA) lysis buffer and protease inhibitors (Applygen Technologies, China) to extract proteins. The protein extraction protocol has been previously described [19]. Primary antibodies including anti-CTSK (1:500; LifeSpan BioSciences, USA), anti-NFATc1 (1:1000; Cell Signaling Technology, USA), and anti-SOCS1 (1:2000; Affinity Biosciences, USA) were used. β-actin (1:50000; Abclonal, China) was used as a control.

### Immunohistochemistry (IHC)

For IHC analysis, paraffin-embedded sections were successively deparaffinized in xylene, dehydrated in ethanol, and incubated in citrate buffer (MXB Biotechnologies, Fuzhou, China) for 10 min at 90 °C for antigen repair. Then, the sections were detected for antigens using a Cell and Tissue staining kit (R&D Systems, Minnesota, USA). Sections were incubated at 4 °C with anti-SOCS1 (1:200; Affinity Biosciences, USA) staining overnight. Images were obtained using a fluorescence microscope system (OLYMPUS, Tokyo, Japan).

### Statistical analysis

Statistical analysis was performed using SPSS 22.0 software. Student’s t-test was used to determine statistical significance. A p-value less than 0.05 was considered to indicate a significant difference.

## Results

### The expression of miR-155 in BMMNCs was significantly decreased during osteoclast induction

**Fig. 1:**
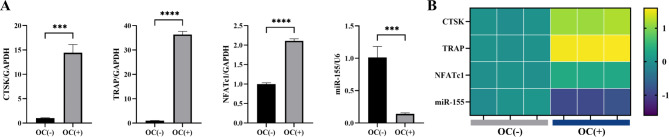
Compared with the BMMNCs without OC induction, the expression of miR-155 in BMMNCS after 3 days of induction showed an opposite trend with osteoclast-related markers. **A** The expression of osteoclast-related markers, including CTSK, TRAP, and NFATc1, was up-regulated in OC-treated cells, as detected by RT-PCR (p < 0.05), while miR-155 expression was significantly down-regulated (p < 0.05). **B** The RT-PCR results were visualized as a heat map to analyze the expression patterns, revealing the inverse trend of miR-155 compared to the other three genes. Student’s t-test, n = 3, ***P < 0.001, ****P < 0.0001

To investigate the role of miR-155 in the osteoclast differentiation of monocytes, BMMNCs from mice were treated with M-CSF (30ng/ml) and RANKL (100ng/ml) to induce osteoclast formation. The expression of osteoclast-related markers, including Cathepsin K (CTSK), TRAP, and Nuclear factor of activated T-cells, cytoplasmic 1 (NFATc1), was examined by RT-PCR. Additionally, the expression of miR-155 was also evaluated. The results showed that compared with BMMNCs without induction, the expression of osteoclast-related markers (CTSK, TRAP, NFATc1) was significantly increased, while the expression of miR-155 was significantly decreased after 3 days of osteoclast induction. These findings indicate an inverse relationship between miR-155 expression and osteoclast-related indicators (Fig. 1).

### Impact of miR-155 overexpression or suppression on osteoclast differentiation ability of BMMNCs

**Fig. 2:**
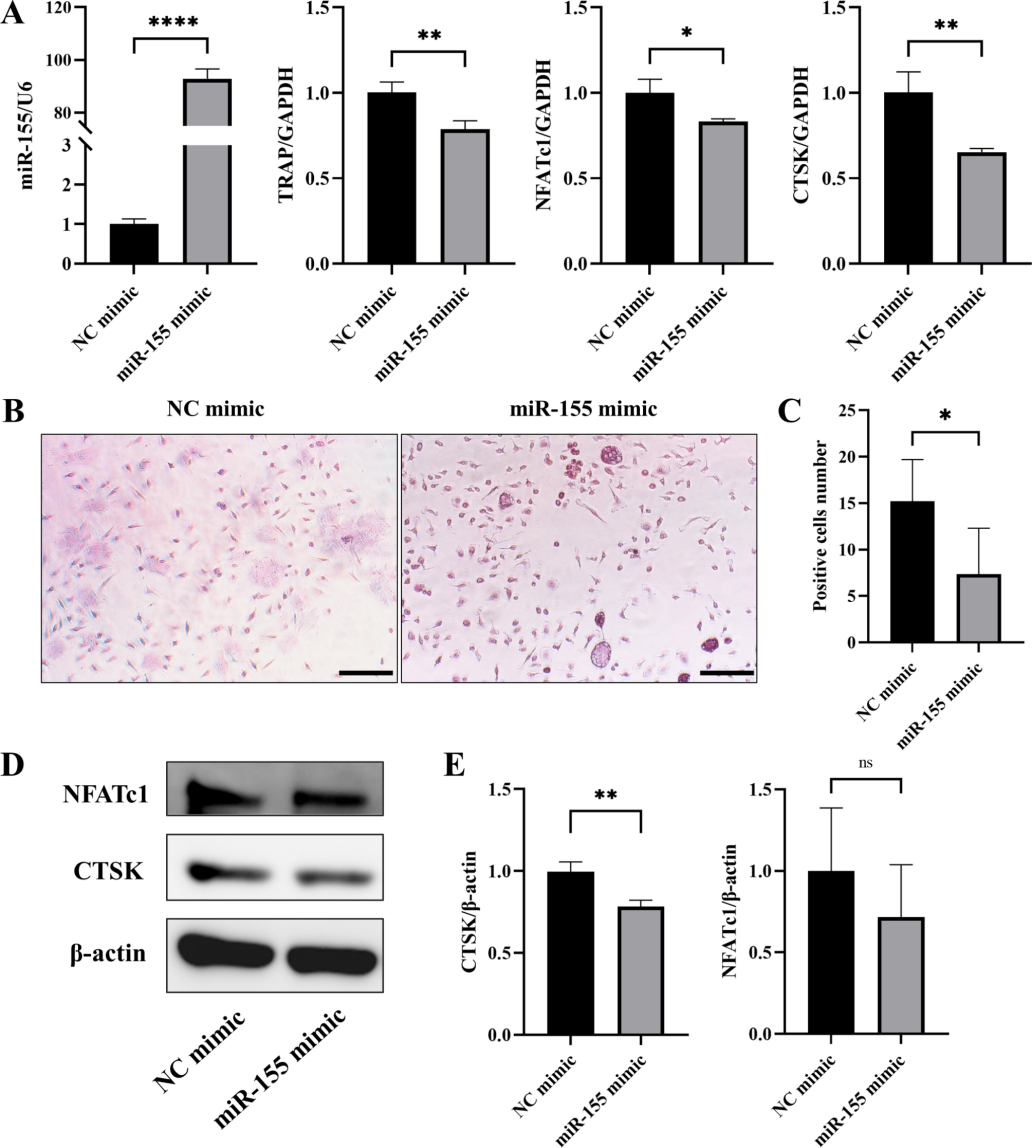
After transfection with miR-155 mimic, the expression of osteoclast-related markers decreased. **A** The expression of osteoclast-related markers, including CTSK, TRAP, and NFATc1, was observed to decrease significantly in miR-155 mimic transfected cells (p < 0.05). **B-C** TRAP staining was performed on cells induced for osteoclast after 5 days of transfection, and a notable reduction in the number of TRAP-positive cells was observed. TRAP-positive cell area was quantified by Image J, the scale bars are 100 μm. **D-E** Western blotting showed that the expressions of osteoclast-related proteins CTSK and NFATc1 decreased after 5 days of osteoclast induction. Image J was used for densitometric analysis measurements. Student’s t-test, n = 3, *P < 0.05, **P < 0.01, ****P < 0.0001

To further investigate the impact of miR-155 on osteoclast differentiation, we employed miR-155 mimic to achieve miR-155 overexpression. The control group was treated with NC mimic at a final concentration of 50nM. Osteoclast induction was conducted 48 h after transfection. After 3 days of induction, RT-PCR analysis revealed a significant increase in miR-155 expression, along with a notable decrease in the expression of osteoclast-related markers, namely TRAP, NFATc1, and CTSK. These differences were statistically significant (Fig. 2A). Both groups of cells underwent osteoclast induction for 5 days, followed by TRAP staining. The results demonstrated a lower number of TRAP-positive cells in the miR-155 mimic group compared to the NC mimic group, with statistical significance (Fig. 2B-C). Western blot analysis further indicated decreased levels of NFATc1 and CTSK, both being osteoclast-related proteins, in the miR-155 mimic group (Fig. 2D-E). Collectively, these findings suggest that miR-155 overexpression inhibits the osteoclast differentiation ability of monocytes.

**Fig. 3:**
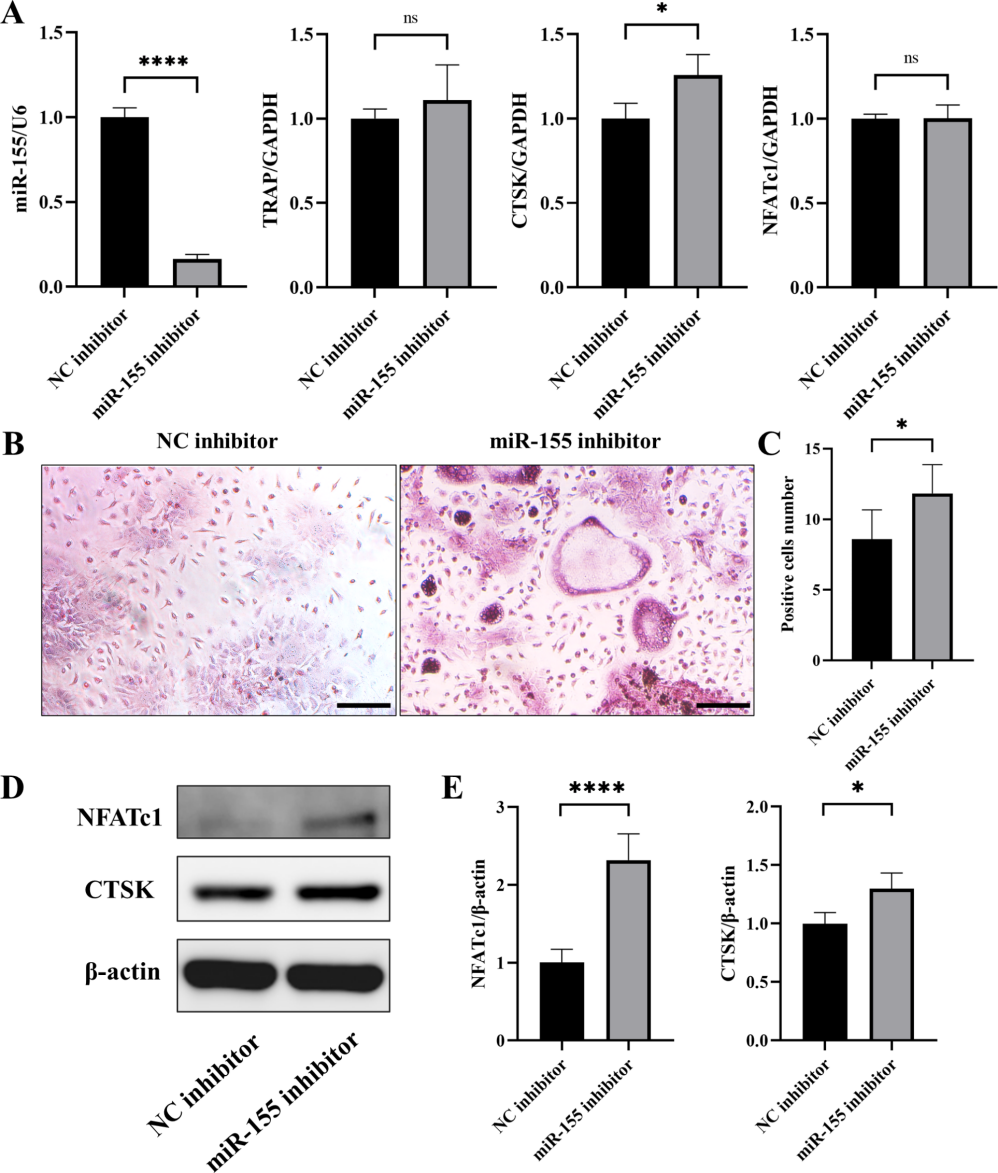
After transfection with miR-155 inhibitor, the expression of osteoclast-related indicators increased. **A** The expression of the osteoclast-related marker CTSK was observed to increase, while miR-155 was decreased in cells transfected with the inhibitor reagent for 48 h followed by osteoclast induction for 3 days (p < 0.05). However, there was no significant difference in the expression of TRAP and NFATc1. **B-C** TRAP staining was performed on cells induced for osteoclast for 5 days after transfection, and a notable increase in the number of TRAP-positive cells was observed. TRAP-positive cell area was quantified by Image J, the scale bars are 100 μm. **D-E** Western blotting showed that the expressions of osteoclast-related proteins CTSK and NFATc1 were increased after 5 days of osteoclast induction. Image J was used for densitometric analysis measurements. Student’s t-test, n = 3, *P < 0.05, ****P < 0.0001

Simultaneously, we utilized a miR-155 inhibitor to induce low expression of miR-155, while the control group was treated with NC inhibitor at a final concentration of 100nM. Similarly, the cells underwent a 48-hour transfection period before osteoclast induction. After 3 days of induction, RT-PCR analysis revealed a decrease in miR-155 expression. Additionally, the osteoclast-related markers TRAP and CTSK showed a tendency to increase, while the expression of NFATc1 did not exhibit a significant difference (Fig. 3A). Following 5 days of osteoclast induction, TRAP staining was performed on the cells of both groups, indicating a substantial increase in the number of TRAP-positive cells in the miR-155 inhibitor group. The difference in the number of positive cells was statistically significant (Fig. 3B-C). Furthermore, Western blotting demonstrated upregulation in the expressions of the osteoclast-related proteins NFATc1 and CTSK in the miR-155 inhibitor group (Fig. 3D-E). These results collectively suggest that the ability of monocytes to differentiate into osteoclasts is enhanced when miR-155 expression is reduced.

### Effect of miR-155 antagonist injection on orthodontic models

**Fig. 4:**
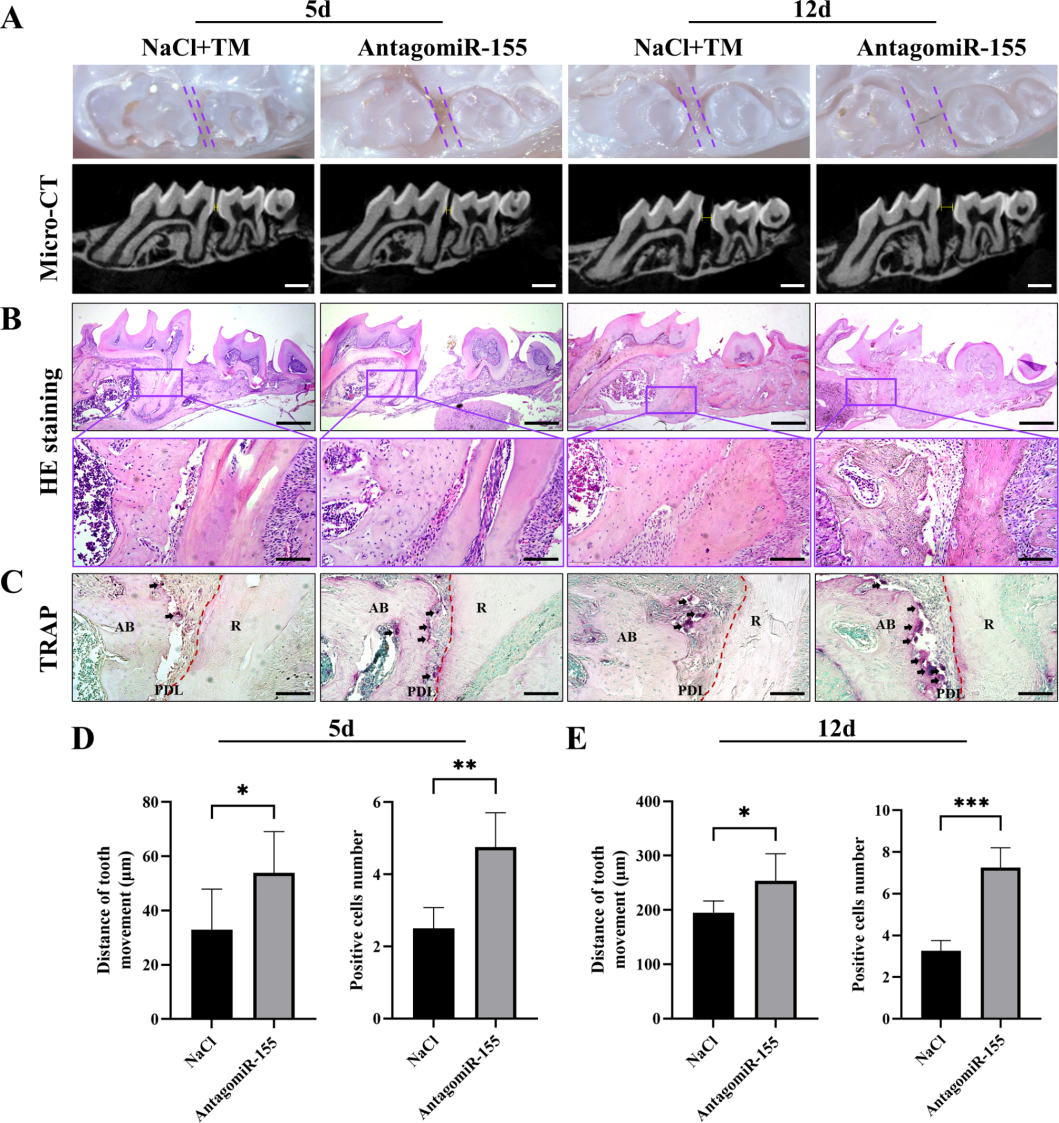
Inhibition of miR-155 accelerates orthodontic tooth movement in mice. **A** Following local injection of NaCl or AntagomiR-155 around the moving teeth of mice, the tooth movement and tissue changes around the teeth were examined using stereoscopic microscopy and micro-CT on the 5th and 12th day. Notably, mice in the AntagomiR-155 group exhibited a faster rate of tooth movement. The scale bars are 500 μm. **B** HE staining was used to observe the tooth movement and bone remodeling in the pressure side of the distal root of the first molar. The scale bars of the first row were 500 μm, and the second row was 100 μm. **C** The number of osteoclasts in the pressure side of the distal root of the first molar was observed by TRAP staining; positive cells were shown in purplish red under the microscope, and black arrows indicated positive cells. The scale bars are 100 μm. R = root, PDL = periodontal ligament, AB = alveolar bone. **D-E** Tooth movement distance was quantified using micro-CT on the 5th and 12th days. The measurement involved determining the shortest distance between the crowns of the first and second molars in the sagittal direction. TRAP-positive cells were also counted. Statistical analysis showed that the AntagomiR-155 group had a longer tooth movement distance and a higher number of osteoclasts at both time points. Student’s t-test, n = 6, *P < 0.05, **P < 0.01, ***P < 0.001

Based on the above results, it is evident that the presence of miR-155 significantly influences osteoclast differentiation. Specifically, when miR-155 is expressed at low levels, there is an increase in osteoclast differentiation-related indicators. To investigate this further, an orthodontic tooth movement model was established in mice. AntagomiR-155 was injected around the right upper first molar of the mice, while the control group received a normal saline injection. Stereoscopic microscopy, micro-CT, HE staining, and TRAP staining were employed to evaluate the outcomes. Remarkably, the AntagomiR-155 group exhibited accelerated tooth movement, regardless of the duration (5 days or 12 days) (Fig. 4A, D-E). At the same time, HE staining showed active remodeling of the alveolar bone and some bone resorption lacunae on the pressure side of the distal root of the first molar (Fig. 4B). To further clarify the activity of osteoclasts, TRAP staining was used to observe that the number of TRAP-positive cells in the AntagomiR-155 group was significantly increased both on day 5 and day 12 (Fig. 4C-E). These findings suggest that local inhibition of miR-155 in vivo during orthodontic force application on periodontal tissue induces a more active osteoclast response, leading to enhanced bone remodeling and increased rate of tooth movement.

**Fig. 5:**
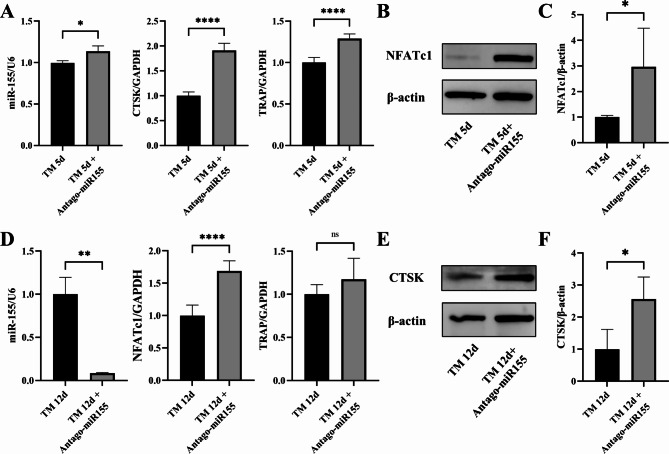
AntagomiR-155 increased osteoclast-related markers in gingival tissues. **A** The expression of miR-155, as well as osteoclast-related markers CTSK and TRAP, was found to be increased in the AntagomiR-155 group (TM 5d + AntagomiR-155) compared to the orthodontic group (TM 5d). **B-C** Western blotting analysis revealed that TM 5d + AntagomiR-155 resulted in an upregulation of the osteoclast-related protein NFATc1 in gingival tissues. **D** At the other time point, the AntagomiR-155 group (TM 12d + AntagomiR-155) showed significantly decreased miR-155 expression and increased osteoclast-related marker NFATc1 expression compared with the orthodontic group (TM 12d). There was also a tendency for increased TRAP expression. **E-F** Western blotting showed that TM 12d + AntagomiR-155 increased the expression of osteoclast-related protein CTSK in gingival tissues. Student’s t-test, n = 3, *P < 0.05, **P < 0.01, ****P < 0.0001

We collected gingival tissue surrounding the right upper first molar from different groups of animal models and performed RT-PCR to analyze gene expression. On the fifth day of force application, the AntagomiR-155 group (TM 5d + AntagomiR-155) exhibited increased expression of miR-155 compared to the orthodontic group (TM 5d), along with elevated expression of osteoclast-related markers CTSK and TRAP (Fig. 5A). Total protein was extracted from the ground gingival tissues for Western blotting, which demonstrated a significant increase in the osteoclast marker NFATc1 in the TM 5d + AntagomiR-155 group (Fig. 5B-C). Similarly, PCR was performed on gingival tissues from the TM 12d + AntagomiR-155 group, revealing a significant decrease in miR-155 expression and an increase in osteoclast-related markers NFATc1 and TRAP (Fig. 5D). Western blotting results showed an elevated expression of CTSK protein in the TM 12d + AntagomiR-155 group, consistent with the 5-day trend (Fig. 5E-F). These findings suggest that the miR-155 inhibitor enhances the expression of osteoclast-related markers, thus influencing orthodontic tooth movement. The results obtained from both in vivo and in vitro experiments are in good agreement.

### MiR-155 directly targets SOCS1 to affect osteoclast differentiation

**Fig. 6:**
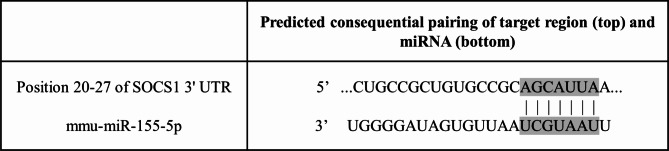
TargetScan software was utilized to predict the binding sites between miR-155 and SOCS1. The software prediction results revealed the presence of target binding sites between miR-155 and SOCS1 in the 3’UTR region

**Fig. 7:**
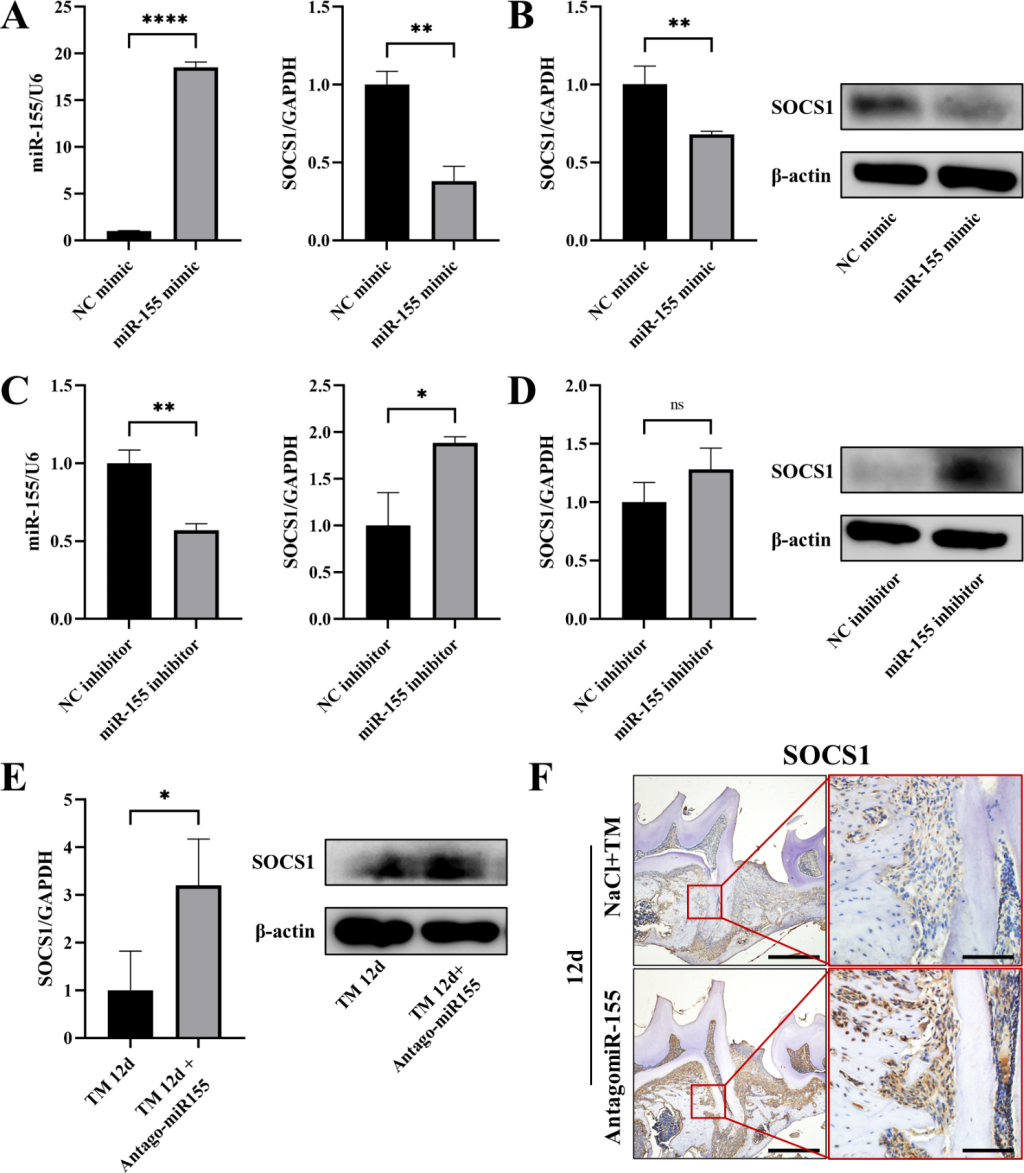
SOCS1 is a potential binding target of miR-155 in osteoclasts. **A** Following transfection of BMMNCs with miR-155 mimic reagent for 48 h, miR-155 expression significantly increased, while SOCS1 expression markedly decreased, demonstrating an inverse correlation. **B** After osteoclast induction, RT-PCR and Western blotting results showed that the expression of SOCS1 in BMMNCs transfected with miR-155 mimics was significantly decreased, which was consistent with the changing trend of osteoclast-related markers. **C** Conversely, transfection of miR-155 inhibitor into BMMNCs resulted in a notable reduction in miR-155 expression and a significant increase in SOCS1 expression after 48 h. **D** After osteoclast induction, RT-PCR and Western blotting results showed that the expression of SOCS1 in BMMNCs transfected with miR-155 inhibitor was increased. **E** RT-PCR and Western blotting analysis of gingival tissue surrounding the moving teeth on day 12 revealed elevated expression of SOCS1 in the AntagomiR-155 group. **F** Immunohistochemical results showed that the AntagomiR-155 group had a significant increase in SOCS1 positive area in the periodontal ligament and bone resorption lacunae on the pressure side of orthodontic tooth movement at 12 days. The scale bars of the first column were 500 μm, and the second column were 100 μm. Student’s t-test, n = 3, *P < 0.05, **P < 0.01, ****P < 0.0001

TargetScan software was utilized to predict the binding sites between miR-155 and suppressor of cytokine signaling 1 (SOCS1), confirming the presence of target binding sites in the 3’ UTR region of SOCS1 (Fig. 6). Meanwhile, previous studies confirmed that miR-155 directly inhibited the 3’UTR region of SOCS1 in luciferase assay [20]. To further explore whether miR-155 targeted SOCS1 plays a role in orthodontic tooth movement and osteoclast differentiation, RT-PCR was performed to analyze the expression pattern of SOCS1 under miR-155 transfection conditions both in vitro and in animal models. BMMNCs were transfected with a miR-155 mimic to achieve miR-155 overexpression. After 48 h, RNA was extracted to assess the transfection efficiency. The results demonstrated a significant increase in miR-155 expression and a corresponding decrease in SOCS1 expression, indicating the direct inhibitory effect of miR-155 on SOCS1 (Fig. 7A). Following osteoclast induction after miR-155 overexpression, the expression of SOCS1 remained significantly reduced (Fig. 7B).

Conversely, a miR-155 inhibitor was employed to downregulate miR-155 expression, and the transfection efficiency was verified after 48 h. The findings revealed a substantial decrease in miR-155 expression in the miR-155 inhibitor group, accompanied by an opposite trend in SOCS1 expression, showing a significant increase (Fig. 7C). After osteoclast induction in the miR-155 inhibitor group, the expression of SOCS1 continued to exhibit an increasing trend (Fig. 7D). These results indicate that the expression of SOCS1 is under the targeted control of miR-155 in vitro, and the change in SOCS1 expression is inversely correlated with miR-155 and consistent with other osteoclast-related markers.

In addition, the expression of SOCS1 in gingival tissue from the animal model was explored. The results demonstrated a statistically significant difference observed on the 12th day, the expression of SOCS1 was significantly increased in the AntagomiR-155 group (Fig. 7E). Immunohistochemical results showed that the AntagomiR-155 group had a significant increase in SOCS1 positive area in the periodontal ligament and bone resorption lacunae on the pressure side of orthodontic tooth movement at 12 days (Fig. 7F).

Overall, these findings provide evidence that miR-155 directly targets SOCS1 to modulate osteoclast differentiation both in vitro and in vivo. The expression pattern of SOCS1 is inversely regulated by miR-155, consistent with the expression of other osteoclast-related markers.

## Discussion

MiRNAs play a crucial role as post-transcriptional regulators in maintaining bone homeostasis. In our study, we initially observed an inverse relationship between miR-155 expression and osteoclast-related markers during osteoclast differentiation, suggesting that inhibition of miR-155 may enhance osteoclast activity. This study is the first to directly manipulate miR-155 expression in BMMNCs using both overexpression and low expression reagents, allowing for positive and negative verification and obtaining relatively stable in vitro experimental results.

To further investigate the impact of miR-155 on osteoclast differentiation, we designed a mouse orthodontic tooth movement model. Osteoclast activation is a critical step in orthodontic tooth movement, and therefore, we examined the effect of miR-155 on this process. Following the injection of the miR-155 inhibitor, we observed an accelerated tooth movement rate, providing further confirmation of our hypothesis that miR-155 acts as an inhibitor of osteoclasts.

The average duration of orthodontic treatment in clinical practice is approximately 20 months. However, as the treatment time extends, both doctors and patients may encounter increased challenges and risks. These include problems such as root resorption, tooth demineralization, and even dental caries [21]. Therefore, accelerating orthodontic tooth movement and reducing treatment time have become areas of active research. Currently, the main methods for acceleration can be categorized into three groups: surgical approaches, physical therapies, and pharmacological interventions [22]. In clinical practice, one commonly used method for accelerating orthodontic tooth movement is corticotomy, which has evolved into a technique known as periodontal accelerated osteogenic orthodontics (PAOO). However, surgical procedures like PAOO are invasive, expensive, and may not be suitable for all patients seeking to accelerate tooth movement [23]. Physical therapy for accelerating orthodontic tooth movement includes the use of direct current devices and low-level laser therapy (LLLT). Originally employed for pain relief and promoting soft tissue healing, LLLT has shown potential in expediting tooth movement. Cruz et al. applied LLLT to orthodontic patients for the first time and confirmed its ability to accelerate tooth movement and reduce treatment time [24]. However, the short observation periods in existing experiments limit our understanding of the exact efficacy and potential side effects of LLLT. As a result, LLLT has not yet gained widespread use in clinical practice [25, 26]. In the realm of drug treatment, Chang et al. introduced a novel sustained-release agent for RANKL [27]. This agent, when released, demonstrated a positive influence on the induction of osteoclast differentiation in RAW 264.7 cells. Moreover, the injection of this sustained-release RANKL facilitated accelerated orthodontic tooth movement. Currently, research on drug treatment primarily relies on animal experiments, aiming to promote osteoclast differentiation and expedite orthodontic tooth movement. However, the long-term stability of these approaches remains uncertain.

Previous studies have indicated that miRNAs possess several favorable characteristics, including their small molecular weight, charge, good water solubility, and rapid degradation and renal metabolism within the body [28]. Based on these characteristics, miRNAs are considered to have a high level of biological safety. Previous studies have established a link between miR-155 and bone metabolism. Transfection of miR-155 mimic has been shown to significantly suppress osteoclast formation, while transfection of miR-155 inhibitor has been observed to significantly increase osteoclast formation. These findings provide evidence for the inhibitory effect of miR-155 on osteoclast differentiation [16]. Meanwhile, miRNA-155 has also been identified as a negative regulator of osteogenic differentiation. In in vitro studies, miRNA-155 was found to inhibit the osteogenic differentiation of mesenchymal stem cells (MSCs) induced by bone morphogenetic protein 9 (BMP-9). This inhibitory effect was attributed to the suppression of Smad/BMP signaling activity and the downregulation of Runt-related transcription factor 2 (Runx2) and bone morphogenetic protein receptor 2 (BMPR2) expression. In in vivo experiments, the injection of a miRNA-155 agonist resulted in the inhibition of ectopic bone formation [29].

In this study, we utilized 6-week-old C57BL/6 male mice as the experimental subjects to establish an orthodontic tooth movement model and investigate the role of miR-155 in vivo. Local injection of AntagomiR-155 at the recommended concentration, as stated in the product manual, was found to accelerate orthodontic tooth movement, potentially through the promotion of local osteoclast activity. MiR-155 may be implicated in the inhibition of osteoclast differentiation. Nonetheless, it is important to acknowledge the limitations of this study. For instance, the duration of the in vivo experiment was relatively short, and the investigation did not extend to the assessment of alveolar bone remodeling and tooth stability following tooth movement. Jiang et al. demonstrated a decrease in miR-155 expression in the gingival crevicular fluid of orthodontic patients as the degree of root resorption increased [17]. However, the assessment of root resorption was not performed in this particular experiment with mice. This aspect could be further investigated in future studies. Additionally, the mechanism underlying the relationship between miR-155 and orthodontic tooth movement requires further elucidation and refinement.

Regarding the investigation of miR-155 target genes, we initially identified a target binding site between the 3’UTR region of SOCS1 and miR-155 using TargetScan software. Subsequently, we performed RT-PCR and western blot to examine the changes in SOCS1 expression upon transfection of cells with miR-155 overexpression or low expression, and we used the Immunohistochemistry to observe the changes of SOCS1 around the orthodontic teeth after injection of miR-155 inhibitor. The results revealed that the expression of SOCS1 exhibited an opposite trend to that of miR-155, consistent with the expression pattern of osteoclast-related markers, and the immunohistochemistry result in this study verified the spatial expression of SOSC1 in the tissues during the tooth movement. Suppressor of cytokine signaling 1 (SOCS1) serves as a critical negative regulator for various inflammatory cytokines. It functions as the most potent member of the SOCS family, exerting negative regulation on the JAK/STAT pathway, and plays a crucial role in suppressing the secretion of proinflammatory cytokines such as interferon-γ (IFN-𝛾) [30]. The JAK/STAT signaling pathway is primarily regulated by cytokines and serves as a key regulator of innate immunity and the modulation of adaptive immune mechanisms. It plays diverse roles in various diseases, including rheumatoid arthritis (RA), Parkinson’s disease (PD), multiple sclerosis, sepsis, and tumors. This pathway is instrumental in controlling inflammation and immune responses [31]. Recent studies have demonstrated that STAT1 plays a crucial role in mediating the inhibitory effect of IFN-γ on osteoclast differentiation [32]. Previous studies have demonstrated that SOCS1 plays a regulatory role in osteoclastogenesis. In vivo, studies using SOCS1-deficient mice have shown that the absence of SOCS1 inhibits LPS-induced osteoclastogenesis and bone destruction compared to wild-type mice [33]. Therefore, the objective of this study is to investigate the targeting of miR-155 on SOCS1 and its impact on osteoclast differentiation. Additionally, the study aims to explore the influence of miR-155 on bone remodeling during orthodontic tooth movement through SOCS1-related pathways, with the goal of providing novel insights into controlling the pace of orthodontic tooth movement in the future. However, this study does have certain limitations. The specific signaling pathways and underlying mechanisms by which miR-155 targets SOCS1 and affects osteoclast differentiation during orthodontic tooth movement require further investigation and will be explored in future research.

## Conclusions

In conclusion, this study has revealed the inhibitory effect of miR-155 on osteoclast differentiation, with enhanced differentiation observed when miR-155 was suppressed. It has been demonstrated that miR-155 directly targets the 3’UTR region of SOCS1, leading to the inhibition of SOCS1 expression and subsequent regulation of osteoclast differentiation. These findings suggest that miR-155 and SOCS1 could serve as potential targets for controlling osteoclast-mediated orthodontic tooth movement.

### Electronic supplementary material

Below is the link to the electronic supplementary material.

Supplementary Material 1

## Data Availability

All data generated or analyzed during this study are included in this published article.

## Abbreviations

MiR-155: MicroRNA-155
MiRNAs: MicroRNAs
3'-UTR: 3’-untranslated region
OSCC: Oral squamous cell carcinoma
OC: Osteoclast induction
BMMNCs: Bone marrow-derived monocytes
PBS: Phosphate Buffered Saline
PS: Penicillin-Streptomycin solution
M-CSF: Macrophage colony-stimulating factor
RANKL: Receptor activator of nuclear factor-κB ligand
TM: Tooth movement
TRAP: Tartrate resistant acid phosphate
HE: Haematoxylin and eosin
RT PCR: Reverse transcriptase polymerase chain reaction
RIPA: Radioimmunoprecipitation
CTSK: Cathepsin K
NFATc1: Nuclear factor of activated T-cells, cytoplasmic 1
SOCS1: Suppressor of cytokine signaling 1
PAOO: Periodontal accelerated osteogenic orthodontics
LLLT: Low-level laser therapy
MSCs: Mesenchymal stem cells
BMP-9: Bone morphogenetic protein 9
Runx2: Runt-related transcription factor 2
BMPR2: Bone morphogenetic protein receptor 2
IFN-𝛾: Interferon-γ
RA: Rheumatoid arthritis
PD: Parkinson’s disease
