# Supplementary material for: MicroRNA-155 targets SOCS1 to inhibit osteoclast differentiation during orthodontic tooth movement
Source: BMC Oral Health. 2023 Dec 1;23:955. doi: 10.1186/s12903-023-03443-8 (PMC10693016; doi:10.1186/s12903-023-03443-8)
Supplement: Supplementary file 1 — Supplementary Material 1 [file 12903_2023_3443_MOESM1_ESM.docx]

**Additional files**

**Figure S1**

**
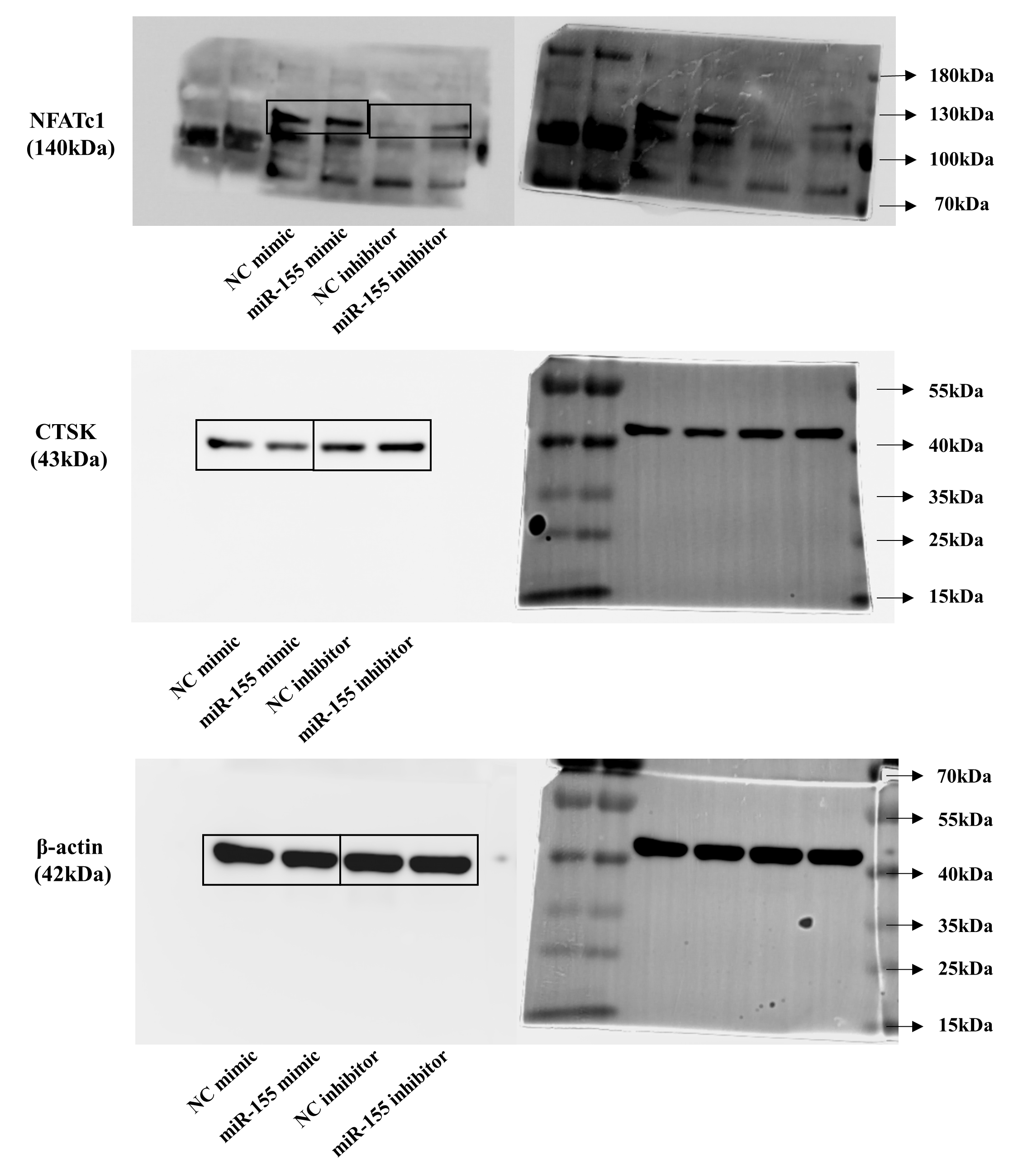
**

**Figure S1.** The complete images of the Western blotting for Figure 2D&3D.

**Figure S2**

**
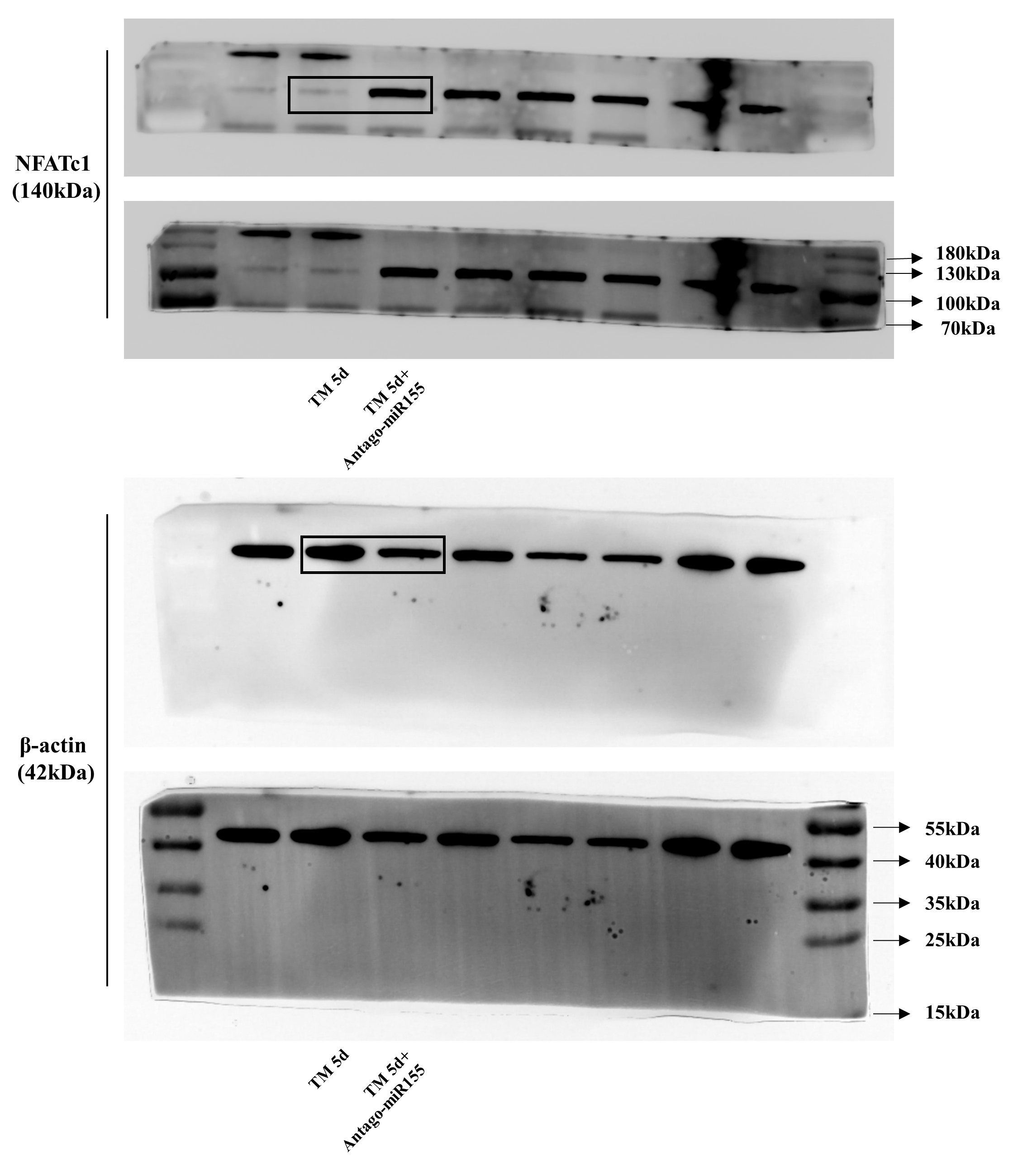
**

**Figure S2.** The complete images of the Western blotting for Figure 5B.

**Figure S3**

**
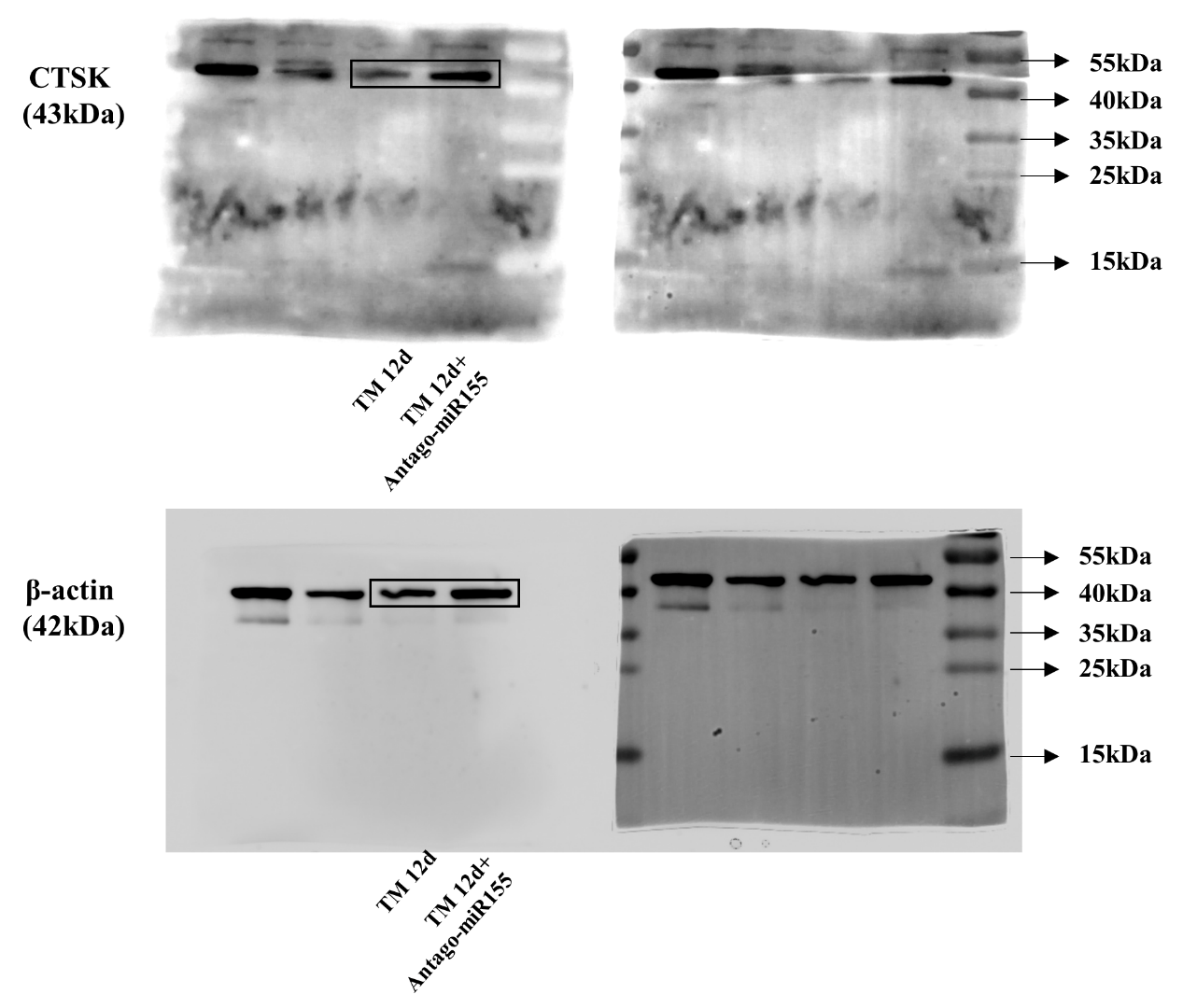
**

**Figure S3.** The complete images of the Western blotting for Figure 5E.

**Figure S4**


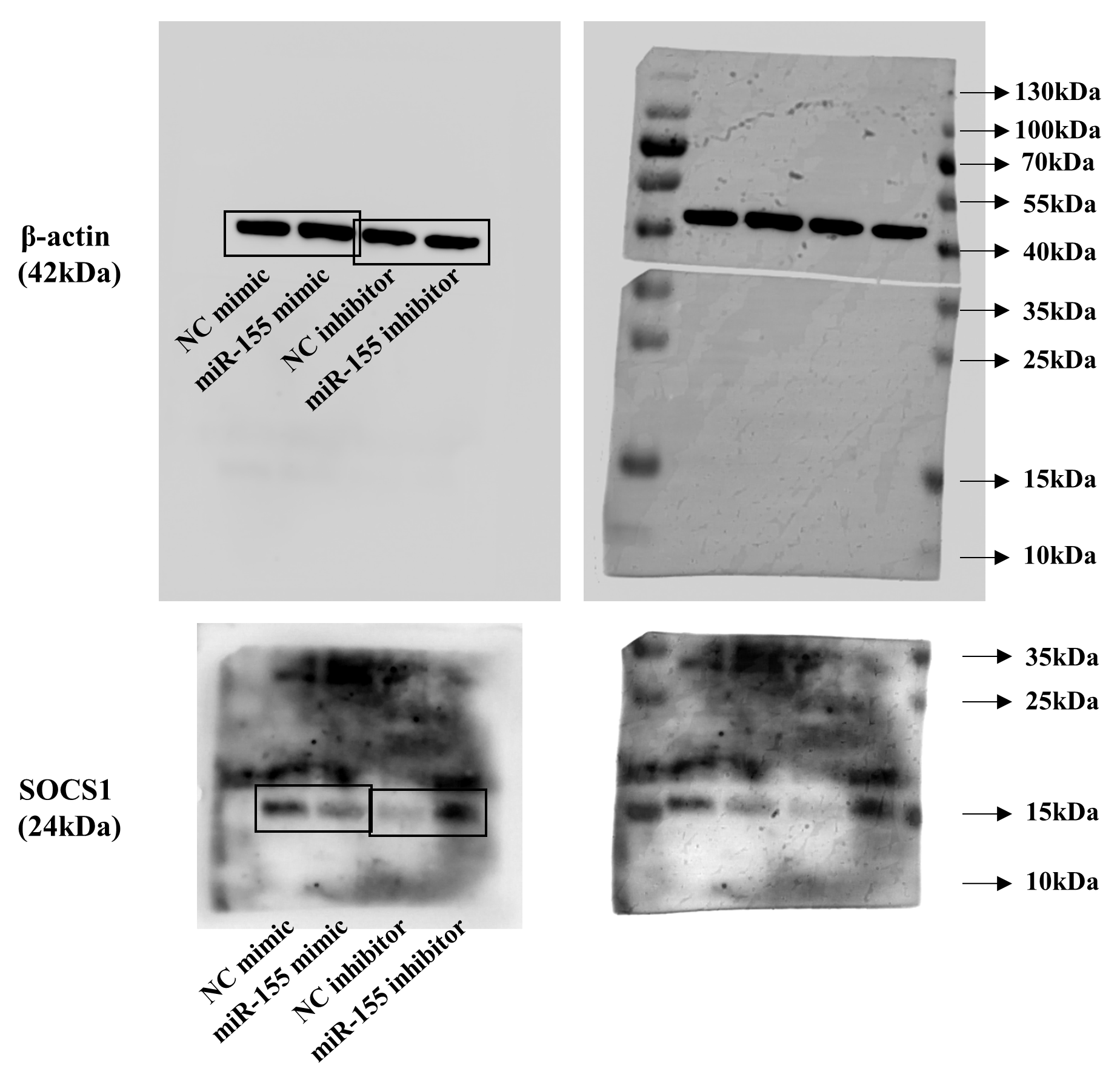


**Figure S4.** The complete images of the Western blotting for Figure 7B, D.

**Figure S5**


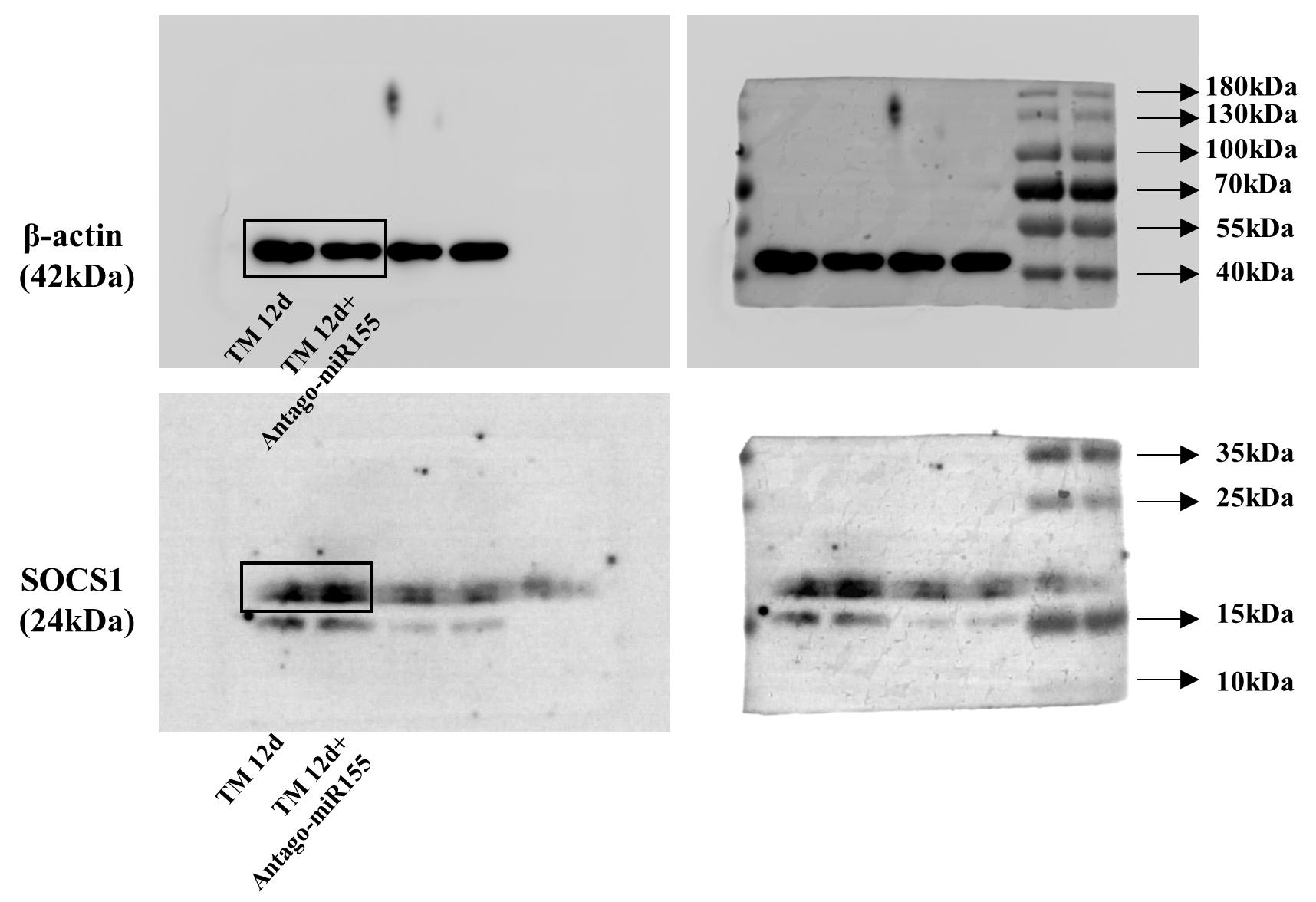


**Figure S5.** The complete images of the Western blotting for Figure 7E.
